# Estrogen Activation by Steroid Sulfatase Increases Colorectal Cancer Proliferation via GPER

**DOI:** 10.1210/jc.2016-3716

**Published:** 2017-09-13

**Authors:** Lorna C. Gilligan, Habibur P. Rahman, Anne-Marie Hewitt, Alice J. Sitch, Ali Gondal, Anastasia Arvaniti, Angela E. Taylor, Martin L. Read, Dion G. Morton, Paul A. Foster

**Affiliations:** 1 Institute of Metabolism and Systems Research, University of Birmingham, Birmingham B15 2TT, United Kingdom; 2 Institute of Applied Health Research, University of Birmingham, Birmingham B15 2TT, United Kingdom; 3 Institute of Cancer and Genomic Sciences, Academic Department of Surgery, University of Birmingham, Birmingham B15 2TH, United Kingdom; 4 Centre for Endocrinology, Diabetes and Metabolism, Birmingham Health Partners, Birmingham B15 2TH, United Kingdom

## Abstract

**Context:**

Estrogens affect the incidence and progression of colorectal cancer (CRC), although the precise molecular mechanisms remain ill-defined.

**Objective:**

The present study investigated prereceptor estrogen metabolism through steroid sulphatase (STS) and 17*β*-hydroxysteroid dehydrogenase activity and subsequent nongenomic estrogen signaling in human CRC tissue, in The Cancer Genome Atlas colon adenocarcinoma data set, and in *in vitro* and *in vivo* CRC models. We aimed to define and therapeutically target pathways through which estrogens alter CRC proliferation and progression.

**Design, Setting, Patients, and Interventions:**

Human CRC samples with normal tissue-matched controls were collected from postmenopausal female and age-matched male patients. Estrogen metabolism enzymes and nongenomic downstream signaling pathways were determined. CRC cell lines were transfected with STS and cultured for *in vitro* and *in vivo* analysis. Estrogen metabolism was determined using an ultra-performance liquid chromatography–tandem mass spectrometry method.

**Primary Outcome Measure:**

The proliferative effects of estrogen metabolism were evaluated using 5-bromo-2′-deoxyuridine assays and CRC mouse xenograft studies.

**Results:**

Human CRC exhibits dysregulated estrogen metabolism, favoring estradiol synthesis. The activity of STS, the fundamental enzyme that activates conjugated estrogens, is significantly (*P* < 0.001) elevated in human CRC compared with matched controls. STS overexpression accelerates CRC proliferation in *in vitro* and *in vivo* models, with STS inhibition an effective treatment. We defined a G-protein–coupled estrogen receptor (GPER) proproliferative pathway potentially through increased expression of connective tissue growth factor in CRC.

**Conclusion:**

Human CRC favors estradiol synthesis to augment proliferation via GPER stimulation. Further research is required regarding whether estrogen replacement therapy should be used with caution in patients at high risk of developing CRC.

## Teaser

We studied how estrogen metabolism affects human colorectal cancer (CRC). We found CRC favored estrogen synthesis, which was proproliferative via a novel G-protein–coupled estrogen receptor pathway.

Controversy surrounds the role estrogens play in the development of colorectal cancer (CRC) (1). Observational studies from the Women’s Health Initiative suggested that premenopausal women have a 20% reduction in CRC compared with age-matched men (2). These sex differences plateau as women become postmenopausal. However, women taking exogenous hormone-replacement therapy (HRT) **(**conjugated estrogen [estrone sulfate (E_1_S)] plus medroxyprogesterone**)** maintain protection against CRC (3). Also, elevated endogenous plasma estrogen concentrations protect against CRC development (4). In contrast, other studies have suggested the greater endogenous plasma estrone (E_1_) concentrations in postmenopausal women increase CRC risk (5). Similarly, women with estrogen-dependent breast cancer have a greater risk of developing CRC (6). Women taking HRT at the time of the diagnosis of CRC are more likely to present with advanced-stage disease (7), suggesting that either the symptoms associated with HRT use leads to a delayed clinical diagnosis or that HRT increases CRC development and proliferative rates.

**Figure 1.**
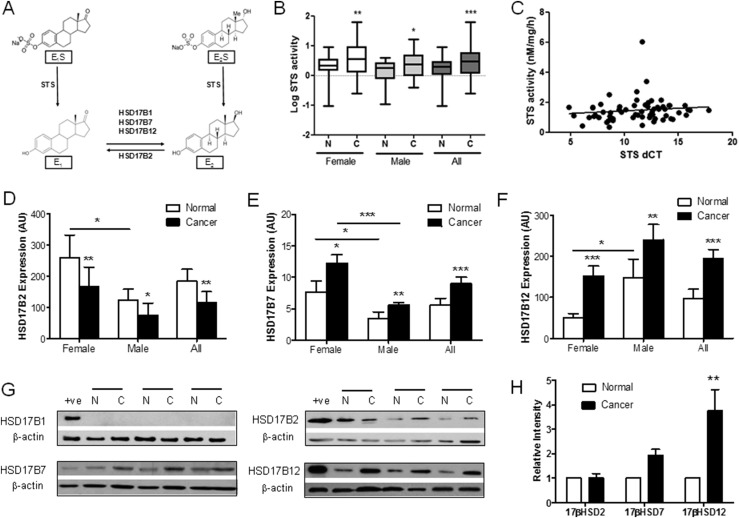
Estradiol synthesis pathways are upregulated in human CRC. (A) Estrogen metabolism pathways demonstrating the importance of STS and HSD17Bs in estrogen synthesis. (B) STS activity is increased in female (n = 29) and male (n = 31) CRC compared with matched normal colon tissue. *P < 0.05, **P < 0.01, ***P < 0.001 using random effects linear regression modeling. (C) STS activity does not correlate with STS expression (dCT) in CRC or normal colon tissue (n = 62). (D) HSD17B2 (female, n = 19; male, n = 28) expression is downregulated in CRC. *P < 0.05, **P < 0.01, ***P < 0.001 (two-tailed paired Student *t* test used). (E and F) HSD17B7 (female, n = 21; male, n = 22) and HSD17B12 (female, n = 21; male, n = 22) expression is upregulated in CRC. *P < 0.05, **P < 0.01, ***P < 0.001 (two-tailed paired Student *t* test used). (G and H) Representative blots and relative intensity (arbitrary unit) of HSD17B enzymes in normal and matched cancerous human colon tissue. HSD17B1 (n = 16) protein expression was not present in human CRC, but HSD17B7 was expressed, and HSD17B12 expression was increased (n = 16), with little change in HSD17B2 expression (n = 16). For relative intensity data, a two-tailed Student *t* test was used. All data presented as mean ± standard deviation.

Because HRT and, thus, estrogens might influence CRC proliferation, the local colonic tissue activation of estrogens via steroid sulfatase (STS) and 17*β*-hydroxysteroid dehydrogenases (HSD17Bs) must be important (8). The expression of STS, the fundamental enzyme desulfating circulating estrogens to their active forms (Fig. 1A), is prognostic for CRC survival (9). Also, messenger RNA (mRNA) expression of HSD17Β2, which catalyzes estradiol (E_2_) to E_1_, is downregulated in human CRC tissue (10), suggesting estrogen metabolism is important in CRC progression. However, little is known about HSD17Β1, HSD17Β7, and HSD17Β12 expression, all of which activate E_1_ to E_2_ (11, 12).

Questions also remain regarding how estrogens act in CRC. Estrogen receptor-*α* (ER*α*) has either low (13) or no (14) expression in both normal colon and CRC, although splice variants do exist (15). Furthermore, loss of the proapoptotic estrogen receptor-*β* (ER*β*), which implies subsequent dominance of other ERs, defines CRC progression (16). A recent meta-analysis has confirmed the loss of ER*β* expression as CRC develops (17). However, no human CRC studies have examined the G-protein–coupled estrogen receptor (GPER), an endoplasmic reticulum membrane-bound receptor with high E_2_-binding affinity (18) and known proproliferative actions in breast (19) and endometrial cancer (20).

We aimed to determine how estrogen metabolism and action affects the development of CRC. By examining key estrogen-metabolizing enzymes in matched normal and cancerous human colorectal tissue and then translating the findings to *in vitro* and *in vivo* systems, we have demonstrated, to the best of our knowledge for the first time, that CRC exhibits dysregulated estrogen metabolism with STS activity and estrogen reductase pathways elevated in CRC. We also found that greater STS activity increases estrogen-stimulated CRC proliferation *in vitro* and *in vivo* through GPER activation via increased expression of connective tissue growth factor (CTGF), a known modulator of GPER action (21). Finally, we have demonstrated that GPER expression is elevated in human CRC tissue, with this significantly correlating with increased CTGF expression. Thus, both STS and GPER inhibition could represent therapeutic targets for patients with CRC.

## Methods

### Experimental procedures

#### Compounds

STX64 (Irosustat) was from Sigma-Aldrich, Ltd. (Dorset, UK) and Professor Barry Potter, University of Oxford. G1 and G15 were from Torcis Bioscience (Abingdon, UK). E_1_S, E_1_, E_2_S, and E_2_ were from Sigma-Aldrich.

#### Human tissue and cell culture

Matched normal and cancerous human colorectal tissue was obtained with local ethics committee approval and informed patient consent. CRC samples from patients with genetic predisposition to CRC, such as familial adenomatous polyposis and hereditary nonpolyposis CRC were excluded. Patients currently receiving HRT were also excluded. The patient characteristics and disease stage are outlined in Supplemental Table 1.

HCT116 and HT-29 cells were cultured in McCoy’s 5a modified medium (Life Technologies, Warrington, UK). Caco2 cells were cultured in minimum essential medium and JEG-3 cells in DM-F12 (Life Technologies). All media were supplemented with 10% fetal bovine serum (FBS) (Sigma-Aldrich) and 2 mM l-glutamine (Sigma-Aldrich). All cell lines were authenticated (March 2014) by short tandem repeat profiling and regularly mycoplasma tested (every 6 months). After 20 passages, the cells were discarded and fresh cells obtained. For all estrogen and GPER antagonist/agonist experiments, charcoal-stripped FBS (sFBS) was used in phenol-free media. Charcoal stripping of FBS is known to reduce estrogen concentrations to undetectable levels.

#### Data sets

Normalized gene expression data generated using the Illuminia RNA-sequencing platform (accessed January 2017) and clinical information were downloaded from cBioPortal (22). The gene expression values were transformed as X = log_2_(X + 1), where X represents the normalized fragments per kilobase transcript per million mapped reads values. Transcriptomic and clinical information were analyzed for 284 patients with colon cancer.

#### Generation of STS overexpressing HCT116 cells

HCT116 cells were transfected using Lipofectamine (Invitrogen, Paisley, UK) with a pCl-neo (OriGene, Cambridge, UK) construct containing either vector only [vo] or complete coding sequence for the human STS [sts] gene. The cells were subsequently grown in 1 mg/mL G418 (Promega, Southampton, UK). STS activity was routinely measured to determine STS transfection stability.

#### STS activity assay

STS activities of human CRC tissue samples and cell lines were measured, as previously described (23). In brief, tissue and cell supernatants were incubated with [6,7-^3^H] E_1_S (4 × 10^5^ dpm; Perkin-Elmer, Coventry, UK) adjusted to a final concentration of 20 μM with unlabeled E_1_S (Sigma). [4-^14^C] E_1_ (1 × 10^4^ dpm; Perkin-Elmer) was included to monitor procedural losses. The samples were incubated at 37°C, after which the product, E_1_, was separated from E_1_S by partition with toluene. ^3^H and ^14^C radioactivity was measured by liquid scintillation spectrometry. The mass of E_1_S hydrolyzed was calculated from ^3^H counts detected and corrected for procedural losses. The results were determined as pmol product formed/h/mg protein.

#### Quantitative reverse transcription polymerase chain reaction analysis

From human samples, 30 mg of tissue was homogenized in RLT buffer containing *β*-mercaptoethanol. Complementary DNA (cDNA) was manufactured using the SENSIFast kit (Bioline) using 1 μg mRNA, as per the manufacturer’s instructions. From the cell lines, mRNA was purified using RNeasy kits (Qiagen, Manchester, UK) as per the manufacturer’s instructions. mRNA samples were reverse transcribed to form cDNA using the Tetro cDNA Synthesis Kit (Bioline Reagents Ltd., London, UK).

Expression of specific mRNAs was determined on a 7500 real-time polymerase chain reaction system (Applied Biosystems) using the QuantiTect Probe reverse transcription polymerase chain reaction kit (Qiagen). Relative expression was determined using the 2^−∆∆Ct^ method. The Taqman assays are described in Supplemental Table 2.

#### Immunoblotting

The blots were probed as outlined in Supplemental Table 3. Secondary antibodies, goat anti-mouse (sc-2005) and goat anti-rabbit (sc-2004), were from Santa Cruz Biotechnologies (Paso Robles, CA). Bound antibody was detected with horseradish peroxidase-conjugated secondary antibody and chemiluminescence. Bands were quantified using ImageJ software from the National Center for Biotechnology Information (available at: http://rsbweb.nih.gov/ij/). The images were converted into binary mode, and ratios were derived by comparing the protein of interest bands to *β*-actin.

#### Liquid chromatography–tandem mass spectrometry

Estrogens were measured using ultra-performance liquid chromatography–tandem mass spectrometry. After the addition of internal standard steroids (E_1_S-d4, E_2_S-d4; Cambridge Isotopes; and ^13C^E_2_; Sigma-Aldrich) samples were extracted using solid phase extraction (C18 Isolute SPE columns, 500 mg; Biotage). Estrogens were quantified relative to a calibration series (0.5 to 500 ng/L) via tandem mass spectrometry. A Waters Xevo mass spectrometer with an electrospray ionization source was used with an attached Acquity liquid chromatography system. Estrogens were eluted from an HSS C18 SB 1.8 µm, 2.1 × 30-mm column using a methanol/water gradient system with 0.3 mM ammonium fluoride added to the aqueous phase. The coefficient of variation for all assays was <20%.

#### Small interfering RNA design and transfection

The small interfering (siRNA) oligonucleotides and transfection reagents were purchased from Dharmacon, Inc. (Lafayette, CO). The predesigned ON-TARGETplus SMARTpool for human GPER and human CTGF genes, containing a mixture of four-targeting siRNA oligonucleotides, was used for knockdown. An ON-TARGETplus Non-Targeting pool, containing four nonspecific siRNA oligonucleotides, was used for control. For siRNA transfection, HCT116 cells were cultured overnight and subsequently transfected with control or GPER or CTGF siRNA oligonucleotides using DharmaFECT transfection reagent, according to the manufacturer’s instructions. The medium was changed to sFBS media every 24 hours and, in certain experiments, included E2 (100 nM) or G1 (100 nM). Proliferation assays were started 48 hours after siRNA transfection.

#### In vivo xenograft studies

Six-week-old athymic, female CD-1 nude mice (*nu−/nu−*) were purchased from Charles River (Margate, UK). All experiments were performed under conditions that complied with institutional guidelines. Five million HCT116 cells were injected subcutaneously into the right flank of the mice. For the STS inhibition studies, when tumors reached 70 to 100 mm^3^, the mice were randomly divided into two treatment groups: oral vehicle (10% ethanol/90% propylene glycol thrice weekly) or oral STX64 (20 mg/kg thrice weekly). For GPER inhibition studies, HCT116[sts]-bearing mice were randomly divided into two treatment groups: intraperitoneal vehicle (0.9% NaCl, 0.1% Tween80, 1% EtOH, thrice weekly) and G15 (50 μg/kg, intraperitoneally thrice weekly). The mice were weighed and tumor measurements taken thrice weekly with the researcher unaware of the groups. The tumor volumes were calculated using the formula (length × width^2^/2). At the conclusion of dosing, the mice were terminated and their tumors removed, weighed, and stored at −80°C.

#### Proliferation assays

Cell proliferation was measured using CyQuant cell proliferation (Thermo Fisher Scientific, Rugby, UK) and 5-bromo-2′-deoxyuridine incorporation assays (Roche Applied Science, Welwyn Garden City, UK), as per the manufacturer’s instructions. Before the experiments, the cells were placed into sFBS phenol-red–free medium (Thermo Scientific) with 5 mM l-glutamine for 72 hours to clear any remaining estrogens in the media. The cells were cultured in flat-bottom 96-well plates in either complete FBS or sFBS phenol-free growth media containing estrogens and subsequent assays performed.

#### Statistical analysis

For human data, the population analyzed was described using summary statistics and relationships between STS activity, and STS expression was investigated by plotting the data and calculating the correlation coefficients. Further analyses used random effects linear regression modeling (with outcomes transformed to reduce the effect of outliers, as appropriate) to allow for patient matching of normal and cancer samples. The models were fitted to investigate differences in STS activity, HSD17B7 mRNA expression, HSD17B12 mRNA expression, and HSD17B2 mRNA expression between the normal and cancer cohorts. For the primary analysis (investigation of differences in STS activity), models were fitted with and without adjustment for patient characteristics (sex, age, and body mass index) and stage (T and Dukes’). For other models, adjustment was made for sex and age. Where model outcomes required log transformation, the estimates obtained were interpreted as approximate percentage differences.

For *in vivo* experiments involving multiple treatment groups, one-way analysis of variance, followed by a Tukey multiple comparison test, was used to determine statistical significance. Where only two groups were compared, Student *t* test was applied. All analysis related to The Cancer Genome Atlas (TCGA) colon adenocarcinoma (COAD) patient survival curves were tested using Kaplan-Meier survival analysis (log-rank method). All statistical analyses were performed using Prism, version 5.0, software.

## Results

### Estrogenic enzymes favor E_2_ metabolism in human CRC

Immunohistochemical studies have shown STS expression is increased in human CRC (9). However, because STS expression does not correlate with enzyme activity and no data are available on STS activity in the human colon, we determined STS activity in human CRC and histopathologically unchanged colonic mucosa located ≥10 to 20 cm away from cancerous lesions (the patient characteristics for 64 participants are listed in Supplemental Table 1). Postmenopausal female and aged-matched male CRC STS activity was significantly increased in CRC tissue compared with the matched tissue (percentage of change, 24.6; 95% confidence interval, 10.3 to 38.8; *P* = 0.001; Table 1, Fig. 1B). Although not formally tested, plotting the data suggested a more pronounced effect in females (Fig. 1B). Increased STS activity did not correlate with increasing STS mRNA expression (dCT) in either normal or cancerous tissue [Fig. 1C; calculated correlation coefficient, 0.27 (*P* = 0.07) and 0.04 (*P* = 0.77) for normal and cancerous samples, respectively]. RNA sequencing data (RNASeq, version 2), analyzed from the TCGA COAD data set showed no substantial change in STS expression from normal to cancerous tissue (Supplemental Fig. 1). STS activity is altered by various post-translational modifications (24), suggesting that determining only STS expression does not represent *in situ* colon activity. Furthermore, STS activity did not correlate with Dukes’ stage or T stage (Table 1), indicating increased STS activity is most likely an early event in tumor formation.

**Table 1.** **Results of Random Effects Linear Models Investigating Differences Between STS Activity and Status (Normal/Cancer), Adjusted for Sex, Age, and BMI**

Variable	Samples (Patients)									
	Unadjusted Model, n = 122 (n = 61)		Adjusted for Sex and Age, n = 122 (n = 61)		Adjusted for Sex, Age, and BMI, n = 84 (n = 42)		Adjusted for Sex, Age, BMI, and T Stage, n = 84 (n = 42)		Adjusted for Sex, Age, BMI, and Dukes’ Stage, n = 84 (n = 42)	
	Change, % (95% CI)	*P* Value	Change, % (95% CI)	*P* Value	Change, % (95% CI)	*P* Value	Change, % (95% CI)	*P* Value	Change, % (95% CI)	*P* Value
Cancer*^a^*	24.8 (12.5 to 37.0)	< 0.001	24.8 (12.5 to 37.0)	< 0.001	24.6 (10.3 to 38.8)	0.001	24.6 (10.3 to 38.8)	0.001	24.6 (10.3 to 38.8)	0.001
Male sex*^b^*			−17.6 (−36.1 to 0.8)	0.061	−21.8 (−37.2 to −6.3)	0.006	−20.6 (−36.4 to −4.7)	0.011	−23.3 (−39.9 to −6.7)	0.006
Age			0.5 (−0.3 to 1.4)	0.266	−0.2 (−0.9 to 0.4)	0.502	0.0 (−0.8 to 0.9)	0.961	−0.3 (−1.0 to 4.7)	0.466
BMI					−0.0 (−1.5 to 1.4)	0.989	−0.1 (−1.6 to 1.4)	0.921	−0.1 (−1.7 to 1.5)	0.876
T stage*^c^*										
2							26.6 (−26.2 to 79.3)	0.323		
3							15.4 (−34.4 to 65.1)	0.546		
4							22.0 (−29.2 to 73.2)	0.400		
Dukes’ stage*^d^*										
B									2.1 (−23.6 to 27.8)	0.872
C									−2.0 (−26.1 to 22.0)	0.867
D									−32.2 (−87.0 to 22.5)	0.249

The outcome used in modeling was log transformed STS activity, and estimates can be interpreted as approximate percentage changes.

Abbreviations: BMI, body mass index; CI, confidence interval.

^a^ Reference was normal.

^b^ Reference was female.

^c^ Reference was stage 1.

^d^ Reference was stage A.

Because STS desulfates circulating and peripheral E_1_S to E_1_, we next determined the expression of enzymes that oxidize E_2_ to E_1_ (HSD17B2) and reduce E_1_ to E_2_ (HSD17Β1, HSD17Β7, and HSD17Β12) in the same human CRC samples. HSD17B2 mRNA was significantly (*P* < 0.01) decreased in CRC tissue compared with that in matched controls (Fig. 1D; Supplemental Table 4; raw dCT values are presented in Supplemental Table 5). HSD17Β1 mRNA was not detectable (data not shown). HSD17Β7 and HSD17Β12 mRNA were significantly increased in female and male CRC tissue compared with matched controls (Supplemental Table 4; raw dCT values are presented in Supplemental Table 5; Fig. 1E and 1F). These data were supported by further analysis of the TCGA COAD data, which demonstrated a substantial decrease in mRNA expression of HSD17B2, and increased expression of HSD17B7 and HSD17B12, in colon cancer (Supplemental Fig. 1). Immunoblotting (Fig. 1G) and subsequent densitometry analysis (Fig. 1H) of normal and cancerous tissue confirmed the lack of HSD17Β1 expression and increased HSD17B12 expression. HSD17Β7 protein expression showed a trend toward increased expression in CRC. In contrast to the mRNA data (Fig. 1F), HSD17Β2 protein was not decreased in CRC compared with the expression in controls (Fig. 1H). HSD17Β4, which oxidizes E_1_ to E_2_, expression was not determined because previous studies have shown this is significantly downregulated in human CRC (25). Taken together, our data suggest that CRC upregulates pathways favoring E_1_S hydrolysis and subsequent E_2_ synthesis.

### Estrogen metabolizing enzyme expression defines CRC estrogenic proliferative response

**Figure 2.**
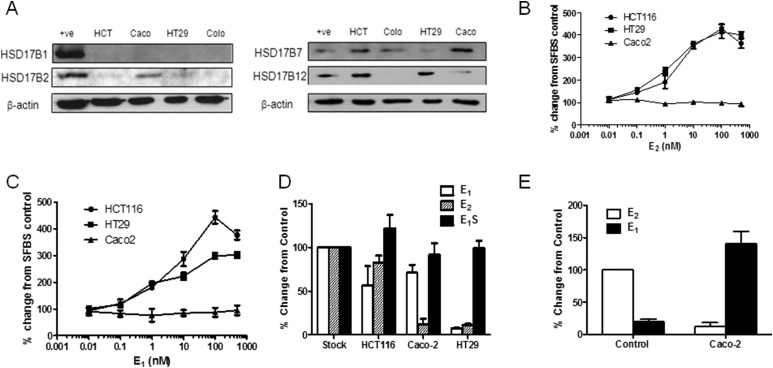
Estrogens increase proliferation in CRC cell lines. (A) Expression profile of HSD17B1, HSD17B2, HSD17B7, and HSD17B12 in HCT116, HT29, Caco-2, and Colo205 cells. *β*-Actin was used as a loading control. One representative blot from three independent experiments. (B and C) E_1_ and E_2_ increased proliferation rates in a dose-dependent manner in HCT116 and HT-29 cells. Caco-2 cells did not respond to E_1_ or E_2_ treatment (n = 4 independent experiments). (D and E) HCT116 cells did not readily metabolize E_1_, E_2_, and E_1_S. HT-29 cells metabolized E_1_ and E_2_ to an unknown metabolite. Caco-2 cells rapidly metabolized E_2_ to E_1_ (n = 3 independent experiments). All data presented as mean ± standard deviation.

Because human CRC exhibited dysregulated estrogen metabolism, we hypothesized that CRC cell lines expressing E_2_ synthesis pathways might be more responsive to estrogen signaling. Thus, we determined the expression patterns of key estrogen-metabolizing enzymes in selected CRC cells. Compared with human CRC tissue, HCT116 and HT-29 cells exhibited similar HSD17Β mRNA (data not shown) and protein (Fig. 2A) expression (*i.e.,* lack of HSD17Β1, presence of HSD17Β7 and HSD17Β12, and limited HSD17Β2 expression). In contrast, Caco2 cells have low HSD17Β7 and HSD17Β12 expression and higher HSD17Β2 expression. Colo205 cells had low or no HSD17B mRNA (data not shown) and protein expression; thus, these cells were not used in further testing. When incubated for 72 hours with E_1_ (Fig. 2B; raw absorbance data shown in Supplemental Fig. 2A) or E_2_ (Fig. 2C; raw absorbance data shown in Supplemental Fig. 2B) in sFBS media, HCT116 and HT-29 cells had increasing dose-dependent proliferative rates compared with the sFBS media controls. The Caco2 cells failed to respond to E_1_ or E_2_ stimulation.

Using liquid chromatography–tandem mass spectrometry, we next examined how CRC cells metabolized estrogens over 24 hours. HCT116 cells did not significantly metabolize E_2_ to other estrogen metabolites, HT-29 cells metabolized E_2_ to unknown metabolites, and Caco2 cells rapidly oxidized E_2_ to E_1_ (Fig. 2D and 2E), indicative of its high HSD17B2 reductase expression. This suggests that oxidation of E_2_ via HSD17Β2, expressed in Caco2 but not in HCT116 and HT-29 cells, affects local E_2_ availability and, consequently, the ability of Caco2 cells to proliferate in response to E_2_. This further implies that peripheral estrogen metabolism in CRC might define the tumors responsiveness to estrogen action.

### STS overexpression augments E_1_S- and E_2_S-stimulated proliferation in CRC

Because STS activity was significantly increased in human CRC samples (Fig. 1B), we examined how overexpression of STS affects CRC proliferation. First, the STS activity of CRC cells was determined (Supplemental Fig. 3A). Caco2 cells had the highest STS activity (165.4 ± 4.7 pmol/mg/h), with HCT116 cells exhibiting very low activity (1.65 ± 0.1 pmol/mg/h). Thus, we selected HCT116 cells to stably transduce with STS (HCT116[sts]) or [vo] (HCT116[vo]). Stable overexpression increased enzyme activity to 200.42 ± 5.91 pmol/mg/h compared with [vo]–expressing controls at 10.58 ± 1.37 pmol/mg/h (Supplemental Fig. 3B).

**Figure 3.**
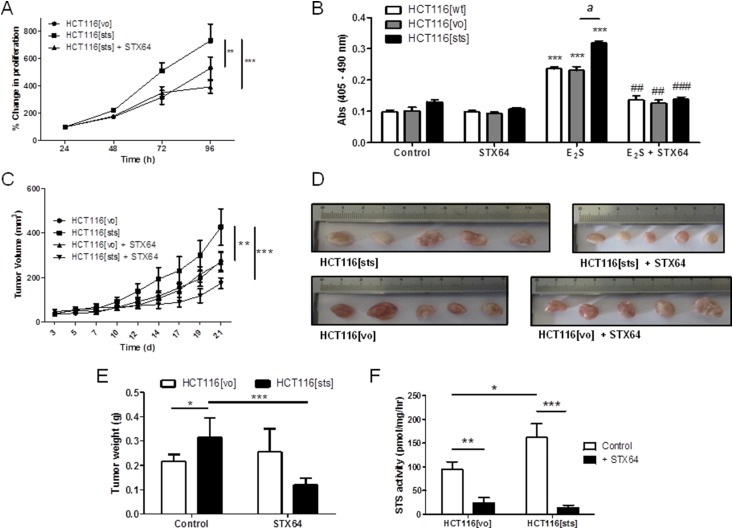
Overexpression of STS in HCT116 cells increased estrogen-dependent proliferation *in vitro* and *in vivo*. (A) HCT116[sts] cells proliferated at a greater rate compared with HCT116[vo] cells. This proliferation was significantly inhibited by STX64 (1 mM). **P < 0.01, ***P < 0.001 (n = 3 independent experiments; one-way analysis of variance, followed by Tukey’s multiple comparison post-test). (B) HCT116[sts] cells had increased proliferation when stimulated with E_2_S (100 nM for 72 hours) compared with wild-type HCT116 (HCT116[wt]) and HCT116[vo] cells. This increased proliferation was blocked by STS inhibition using STX64 (1 mM). ***P < 0.001 compared with control; ##*P* < 0.01, ###*P* < 0.001 compared with E_2_S treatment; *^a^P* < 0.001 compared with HCT116[vo] (two-tailed Student *t* test used; n = 4 independent experiments). (C) HCT116[sts] xenografts grew at an increased rate compared with HCT116[vo] xenografts. This increased proliferation was inhibited by STX64 (20 mg/kg thrice weekly, orally). **P < 0.01, ***P < 0.001 (one-way analysis of variance followed by Tukey’s multiple comparison post-test). (D) Five randomly taken tumors imaged after removal. (E) Wet tumor weights at 21 days after HCT116 cell inoculation. HCT116[sts] resulted in an increased tumor burden, which was inhibited by STX64. *P < 0.05, ***P < 0.001 (two-tailed Student *t* test used). (F) STS activity in HCT116[vo] and HCT116[sts] xenografts at day 21. HCT116[sts] xenograft maintained elevated STS activity compared with HCT116[vo]. STX64 treatment significantly inhibited HCT116[vo] and HCT116[sts] activity. *P < 0.05, **P < 0.01, ***P < 0.001 (n = 5 to 14; two-tailed Student *t* test used). All data presented as mean ± standard deviation.

In full media, HCT116[sts] proliferation significantly increased compared with HCT116[vo] cells (Fig. 3A), with this augmented growth blocked by the noncytotoxic, specific STS inhibitor STX64. Incubation of these same cells in sFBS media supplemented with E_1_, E_2_, or E_1_S (at 100 nM) for 72 hours, a statistically significant (*P* < 0.001) growth difference was observed between HCT116[sts] and HCT116[vo] cells treated with E_1_S only (Supplemental Fig. 3C). This demonstrated greater STS desulfation of E_1_S, leading to increased E_1_ liberation driving proliferation. When these cells were grown for 72 hours in sFBS supplemented with E_2_S (100 nM), all proliferated in response to E_2_S, with the greatest increase seen in HCT116[sts] cells compared with sFBS controls (Fig. 3B). STX64 blocked this increased growth, suggesting estrogen desulfation is an important regulator in CRC proliferation.

### STS overexpression increases CRC xenograft growth

Because HCT116[sts] cells exhibited increased proliferation *in vitro*, we next examined whether this effect was evident in an intact, female mouse CRC xenograft model. HCT116[sts] or HCT116[vo] cells (1 × 10^6^) were subcutaneously implanted into the flanks of female MF-1 nude mice. Intact adult female mice were chosen, because they have circulating E_1_S and E_2_S available for hydrolysis. Over 21 days, HCT116[sts] xenograft growth was significantly (*P* < 0.01) greater compared with the HCT116[vo] controls (Fig. 3C), leading to a greater tumor burden by day 21 after implantation (Fig. 3D and 3E). Dosing of STX64 (20 mg/kg, orally, thrice weekly) initially completely stagnated (days 3 to 18) HCT116[sts] growth (Fig. 3C), although the tumors were proliferating by day 24. Although tumor STS activity was almost completely ablated by STX64 treatment (Fig. 3F), HCT116[vo] xenograft growth was not affected by STS inhibition. This suggests that once STS is overexpressed, CRC might rely more heavily on estrogen desulfation for proliferation.

### Estrogens increase proliferation through GPER signaling in CRC

**Figure 4.**
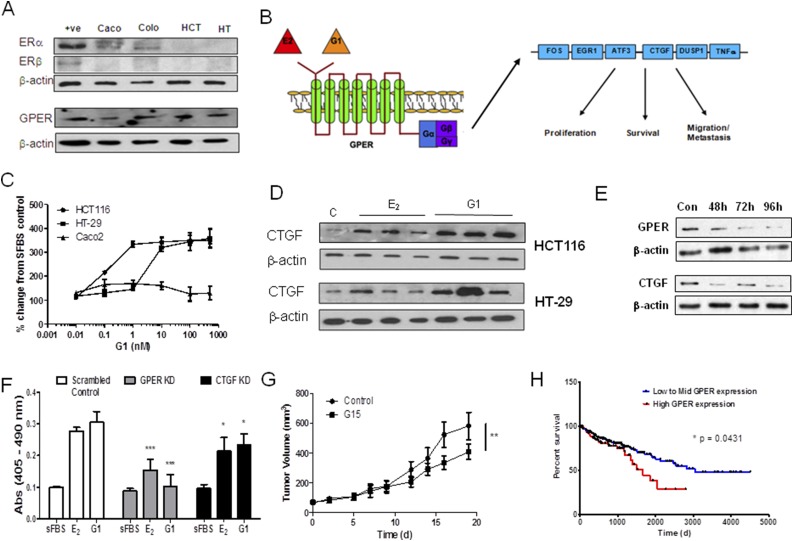
E_2_ acts through GPER signaling to increase CRC proliferation. (A) ER*α* and ER*β* were not expressed in HCT116 or HT-29 but were present in Caco2 and Colo205 cells. GPER was expressed in all cell lines tested. *β*-Actin was used as a loading control. One representative blot from three independent experiments. (B) Schematic of the downstream molecular signaling factors stimulated by GPER action as defined in breast cancer. (C) The GPER agonist G1 increased the proliferation rates in a dose-dependent manner compared with cells grown only in media with sFBS (two-tailed Student *t* test used; n = 4 independent experiments). (C) The GPER antagonist G15 (1 mM) inhibits the increased proliferation induced by E_2_ (100 nM for 72 hours) and G1 (100 nM for 72 hours) in HCT116 and HT-29 cells. **P < 0.01, ***P < 0.001 compared with controls (two-tailed Student *t* test; n = 4 independent experiments). (D) E_2_ (100 nM) and G1 (100 nM) treatment increases CTGF protein expression in HCT116 and HT-29 cells. *β*-Actin was used as a loading control. One representative blot from three independent experiments. (E) siRNA knockdown of GPER and CTGF in HCT116 cells was achieved for 96 hours after siRNA treatment. (F) siRNA knockdown of GPER and CTGF inhibits E_2_ (100 nM) and G1 (100 nM) stimulation of HCT116 proliferation. *P < 0.05, ***P < 0.001 compared with controls (two-way analysis of variance, followed by a Bonferroni post-test; n = 3). (G) G15 (50 mg/kg thrice weekly, intraperitoneally) significantly attenuated HCT116[sts] xenograft tumor growth in female nude mice. **P < 0.01 (two-way analysis of variance; n = 10). (H) Patients with high GPER expression (n = 110) had a significantly worse survival outcome compared with mid to low GPER-expressing (n = 330) CRC tumors, as shown from analysis of the TCGA COAD data set (Kaplan-Meier survival analysis; log-rank method). All data presented as mean ± standard deviation.

Because controversy surrounds how estrogens elicit their effects in CRC (26), we investigated whether GPER was expressed in human CRC and whether GPER stimulation augmented CRC proliferation. In contrast to HCT116 and HT-29 cells, Caco2 and Colo205 cells express ER*α*. None of the CRC cell lines tested expressed ER*β* but all expressed GPER (Fig. 4A). Others (21) have shown in breast cancer that GPER stimulation with E_2_ can increase proliferation and increase the expression of various downstream regulators of survival and migration (Fig. 4B). Thus, we next examined whether the specific GPER agonist G1 (72 hours of treatment) stimulated HCT116, HT-29, and Caco2 cell proliferation, as measured by 5-bromo-2′-deoxyuridine incorporation, compared with sFBS controls (Fig. 4C). G1 induced dose-dependent stimulation in proliferation, with this effect more pronounced in HCT116 and HT-29. These results mimicked the proliferative effects by E_1_ and E_2_ (Fig. 2B and 2C). Intriguingly, Caco2 cells modestly responded to G1 treatment in contrast to their lack of increased proliferation in response to E_2_ (Fig. 2B), supporting the notion that rapid E_2_ oxidization in Caco2 limits estrogenic effects. However, when GPER is stimulated by G1, Caco2 cells can increase proliferation through this pathway. In HCT116 and HT-29 cells, the GPER antagonist G15 (1 μM) blocked both E_2_- and G1-stimulated proliferation over 72 hours compared with the controls (Supplemental Fig. 4A).

To further delineate GPER action in CRC, we also determined how E_2_ and G1 affected downstream molecular regulators of GPER action (21). Figure 4B illustrates the key genes that we examined in CRC cells [*i.e.,* FOS, EGR1, ATF3, CTGF, DUSP1, and tumor necrosis factor-*α* (TNF*α*)]. All these genes were upregulated in response to GPER stimulation in breast cancer cell lines (21). In HT-29 cells, EGR1 (Supplemental Fig. 5A), ATF3 (Supplemental Fig. 5B), DUSP1 (Supplemental Fig. 5C), and CTGF (Supplemental Fig. 4D) but not FOS (Supplemental Fig. 4E) and TNF*α* (Supplemental Fig. 4F) were significantly elevated in response to E_2_ (100 nM for 24 hours) and G1 (100 nM for 24 hours) compared with sFBS-treated cells. In HCT116 cells, EGR1, ATF3, and CTGF were significantly elevated in response to treatment. Because CTGF gave the largest response to E_2_ and G1 stimulation, we further examined its protein expression in response to treatment. In HCT116 and HT-29 cells, E_2_ (100 nM) and G1 (100 nM) increased CTGF protein expression after 24 hours, as measured by immunoblotting (Fig. 4D).

To confirm the importance of GPER or CTGF in mediating the proliferative effects of E_2_, we performed transient knockdown of these two proteins using siRNA and determined their response to E_2_ treatment. In HCT-116 cells, siRNA of GPER and CTGF provided protein knockdown for 96 hours, the period required for subsequent proliferation studies (Fig. 4E). Knockdown of GPER and CTGF significantly (*P* < 0.001 and *P* < 0.05, respectively) inhibited the proliferation driven by E_2_ and G1 in HCT-116 (Fig. 4F). Intriguingly, when we again moved into an *in vivo* model of CRC, the use of the GPER antagonist G15 (at 50 μg/kg intraperitoneally thrice weekly) also significantly (*P* < 0.01) inhibited HCT116[sts] xenograft growth implanted into female nude mice (Fig. 4G). Interrogation of the TCGA COAD data set indicated that although all ERs (ER*α*, ER*β*, and GPER) were significantly (*P* < 0.0001) downregulated in CRC compared with normal controls (Supplemental Fig. 6A), GPER still had the greatest expression in CRC. Further analysis of the TCGA data set demonstrated that patients with CRC tumors expressing high GPER had significantly (*P* = 0.0431) poorer outcomes compared with low to mid-expression levels (Fig. 4H). CRC with high ER*α* expression also resulted in significantly (*P* = 0.0265) worse outcomes (Supplemental Fig. 6C), suggesting the importance of these proproliferative pathways in CRC. High ER*β* expression did not affect CRC patient outcomes (Supplemental Fig. 6D). Regarding CTGF expression, analysis of the TCGA COAD data set showed increased expression in CRC compared with normal colon (Supplemental Fig. 6B). CRC with high mRNA expression of CTGF resulted in a significantly (*P* = 0.0272) poorer outcome (Supplemental Fig. 6E).

**Figure 5.**
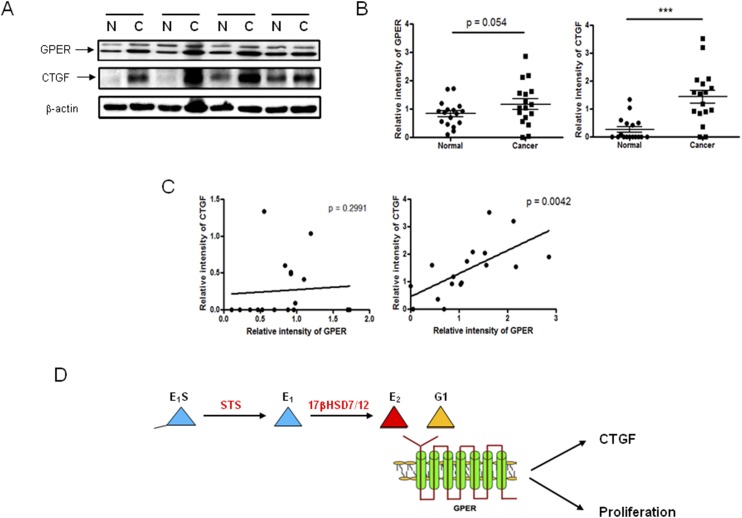
CTGF and GPER expression correlates in human CRC. (A) Immunoblotting of GPER and CTGF expression in normal (N) and cancerous (C) human colon tissue. *β*-Actin was used as a loading control. One representative blot from three independent experiments. (B) GPER and CTGF expression were increased in human CRC, as measured by the immunoblotting relative intensity to *β*-actin. *P < 0.05, ***P < 0.001 (two-tailed Student *t* test used; n = 17). (C) Correlation between GPER and CTGF relative intensity in normal and cancerous human colon tissue (n = 17). (D) Schematic diagram showing proposed pathway through which estrogens act, via GPER, to augment proliferation in CRC.

However, because the correlation of mRNA and protein expression is notoriously poor, hovering at ~40% explanatory power across many studies (27) and GPER protein expression was present in CRC cell lines (Fig. 4A), we determined GPER protein expression in our human CRC samples and demonstrated an almost statistically significant (*P* = 0.054) increase in expression in CRC (Fig. 5A and 5B; Supplemental Fig. 7 shows all original immunoblots). CTGF mRNA (Supplemental Fig. 6B) and protein was significantly (*P* < 0.001) increased in CRC, as determined by relative densitometry (Fig. 5B). The relative intensity of immunoblots for GPER and CTGF highlighted a statistically significant (*P* = 0.0042) and positive correlation between GPER and CTGF expression in cancerous tissue but not in matched normal controls (Fig. 5C). As GPER stimulation increases CTGF expression, our results indicate that greater estrogen availability through STS activity in these tumors might lead to increased GPER stimulation and CTGF expression (Fig. 5D).

## Discussion

We have demonstrated a critical role for prereceptor local estrogen metabolism and action in the proliferation of CRC. We found that estrogen synthesis pathways, via STS, HSD17B7, and HSD17B12, are elevated in CRC and that estrogens stimulate CRC growth through a GPER-mediated mechanism. Of particular importance is STS, a key regulator in estrogen activation. When overexpressed in HCT116 cells, STS drives greater tumor proliferation in *in vitro* and *in vivo* models. Finally, we have demonstrated that E_2_ acts through GPER signaling, most likely via CTGF, in CRC, and that both GPER and CTGF are increased in human CRC. Our results suggest that inhibiting GPER or estrogen metabolism could be a therapeutic option for this malignancy.

Controversy exists on the role of estrogens in CRC development and progression. The Women's Health Initiative (28) has highlighted various questions on how estrogens and progestins affect cancer. Epidemiological studies have indicated estrogens as protective against CRC development. However, how estrogens affect CRC once it has developed is poorly defined. It has been suggested that although initially protective, estrogens might be mitogenic in CRC (26) through changes in local estrogen metabolism and receptor availability. Estrogens promote tumorigenesis in colitis-associated CRC (29), and E_2_ increases LoVo cell line proliferation via upregulation of fatty acid synthesis (30). However, few studies have investigated the enzymes involved in estrogen metabolism in CRC, and the ones that have overlooked key 17*β*HSDs and STS activity. Furthermore, although evidence has strongly suggested that ER*β* downregulation and, thus, the loss of this proapoptotic pathway, is an important turning point in CRC development (16), whether GPER expression or stimulation affects CRC has not previously been determined.

We found that STS activity is significantly elevated in human CRC and that STS overexpression stimulates CRC cell proliferation. Previous findings had indicated that increased STS expression is prognostic for CRC survival (9); however, that study did not measure STS activity. This is an important distinction. STS is subject to post-translational modifications affecting activity. We found that colon STS activity and expression do not correlate. Furthermore, analysis of the TCGA COAD database demonstrated no substantial changes in STS expression in colon cancer compared with normal controls. Although eventual patient outcomes have not yet been determined, we found that STS activity did not correlate with Dukes’ stage or T stage, implying increased STS activity is most likely an early event in CRC development. Thus, its prognostic significance is questionable (9). Along with increased aromatase expression, elevated STS activity is a hallmark of estrogen-dependent cancer (31). STS inhibition is currently in phase II clinical trials of patients with hormone-dependent breast cancer, after it had shown promise in preclinical studies against E_2_S-stimulated breast cancer *in vivo* and in phase I trials (32). Because aromatase expression is not detectable in the human colon (9), local desulfation of circulating E_1_S might act as the primary route for estrogen availability in CRC.

Once desulfated, HSD17Β1, HSD17Β7, and HSD17Β12 reduce E_1_ to E_2_, with HSD17Β2 catalyzing reverse oxidation. Supporting our findings, TCGA COAD analysis and others (25) have shown that HSD17B2 expression is downregulated in CRC; however, our data indicated no change in HSD17Β2 protein expression, suggesting this pathway might remain active. Although HSD17Β1 is the prime reducer of E_1_ (33), we have demonstrated that this enzyme is absent in CRC. HSD17Β7 and HSD17Β12 expression are significantly upregulated in CRC compared with matched normal controls, with this effect mimicked at the protein level, and our findings are supported by the results from the TCGA COAD data analysis. Thus, CRC might favor E_2_ synthesis. Recently, preclinical studies have shown inhibition of HSD17B7 in hormone-dependent breast cancer blocks E_1_ to E_2_ synthesis and thus has therapeutic potential (34). Because intratumoral E_1_ and E_2_ concentrations in CRC tissue pertains to a poor prognosis (9), inhibiting these enzymes in CRC might be therapeutically beneficial.

Because ER*α* and ER*β* are not present in the CRC cell lines tested, the question arose of how estrogens act in CRC. Limited data on colonic GPER expression are available. GPER stimulation might affect colonic motility in mice (35), and its expression might influence abdominal pain severity in inflammatory bowel disease (36). We have demonstrated that GPER protein, but only limited mRNA, is expressed in human CRC tissue and cell lines. GPER protein expression is elevated in human CRC tissue compared with that in matched normal controls, in contrast to mRNA, which is decreased. This might imply that GPER protein degradation pathways are altered in CRC, effectively allowing for GPER protein retention. Stimulation of GPER by E_2_ or the specific agonist G1 increased CRC proliferation *in vitro*, with this effect blocked by GPER inhibition in *in vitro* and *in vivo* CRC models. In contrast to our findings, recent research has shown GPER stimulation by G1 decreases proliferation of various CRC cell lines, including HCT116 (37). However, these studies used higher doses of G1 (≤10 μM) compared with our 100-nM dose. Also, unlike the present work, these studies were not performed in stripped media (*i.e.,* no or low estrogen) conditions. Thus, this finding suggests a biphasic response to G1 and estrogens might be present with regard to GPER stimulation in CRC, with low doses increasing proliferation and high doses inducing apoptosis. This biphasic response is also evident with ER*α* stimulation in breast cancer (38).

GPER deficiency results in multiple physiological alterations, including obesity, cardiovascular dysfunction, insulin resistance, and glucose intolerance (39). Much interest exists in its proproliferative effects in breast cancer. In breast cancer patients, GPER expression has been associated with an increased primary tumor size and the prevalence of distant metastases (40). GPER stimulation by tamoxifen is a potential pathway of tamoxifen-resistant hormone-dependent breast cancer (41). Intriguingly, breast cancer patients treated with tamoxifen are more likely to develop CRC (42). Our results strongly implicate E_2_-GPER–mediated action through CTGF in CRC proliferation. Because the loss of ER*β* defines CRC development (16), it will be of interest to further examine GPER action in the context of ER*β* expression to determine whether an ER “switch” occurs during CRC progression.

Furthermore, in CRC cell lines, the expression of CTGF, a known downstream regulator of GPER action (21), was elevated by E_2_ and G1 treatment. A correlation was evident between GPER and CTGF expression in human CRC tissue. CTGF is upregulated in some CRC patients (43), although its expression is reduced in latter-stage disease (44). Analysis of the TCGA COAD data set also suggested that high CTGF is related to poor patient survival, although others have shown high CTGF expression correlates with improved CRC survival rates (43). This implies a complicated relationship between E_2_ stimulation of GPER, increased proliferation, CTGF-mediated effects, and patient outcomes. However, in general, dysregulation of CTGF expression has been linked to poor outcomes in many human cancers (45).

In conclusion, we have identified an estrogen-driven proliferative pathway in CRC. Increased STS activity leads to greater estrogen desulfation, thereby increasing HSD17B substrate availability for subsequent E_2_ synthesis, followed by GPER activation and CTGF upregulation. These findings identify STS, 17BHSD7, 17BSHD12, and GPER as potential therapeutic targets for CRC.
