# Supplementary material for: Estrogen Activation by Steroid Sulfatase Increases Colorectal Cancer Proliferation via GPER
Source: J Clin Endocrinol Metab. 2017 Sep 13;102(12):4435–47. doi: 10.1210/jc.2016-3716 (PMC5718700; doi:10.1210/jc.2016-3716)
Supplement: Supplementary file 1 [file jc.2016-3716.st1.docx]

| **Characteristic** | **Total n=64** |
| --- | --- |
| **Age (years); median (Q1, Q3) (n=63)** | 72 (63, 81) |
| **Sex (male); n (%) (n=64)** | 33 (52) |
| **BMI (kg/m2); median (Q1, Q3) (n=44)** | 27.0 (24.6, 31.3) |
| **T stage; n (%) (n=63)** |  |
| **1** | 1 (2) |
| **2** | 16 (25) |
| **3** | 30 (48) |
| **4** | 16 (25) |
| **Dukes stage; n (%) (n=63)** |  |
| **A** | 11 (17) |
| **B** | 23 (37) |
| **C** | 27 (43) |
| **D** | 2 (3) |

**Supplementary Table 1:** Characteristics of the population from which CRC tissue obtained
