# Supplementary material for: Estrogen Activation by Steroid Sulfatase Increases Colorectal Cancer Proliferation via GPER
Source: J Clin Endocrinol Metab. 2017 Sep 13;102(12):4435–47. doi: 10.1210/jc.2016-3716 (PMC5718700; doi:10.1210/jc.2016-3716)
Supplement: Supplementary file 2 [file jc.2016-3716.st2.docx]

| **Commercial Taqman Assays** | |
| --- | --- |
| **Gene Target** | **Catalogue Number** |
| STS | Hs00996676_m1 |
| HSD17B1 | Hs00166219_g1 |
| HSD17B2 | Hs00157993_m1 |
| HSD17B7 | Hs00367686_m1 |
| HSD17B12 | Hs00725054_m1 |
| GPER | Hs01922715_s1 |
| CTGF | Hs01026927_g1 |
| FOS | Hs04194186_s1 |
| EGF1 | Hs00228005_m1 |
| ATF3 | Hs00231069_m1 |
| DUSP1 | Hs00610256_g1 |
| TNFa | Hs99999043_m1 |

**Supplemental Table 2:** List of Taqman primers used in study (all from Life Technologies). RT-PCR reactions were performed in a ‘Rotor Gene 2000 Real-Time Cycler’ (Corbett Life Science, Cambridge, UK). Two endogenous control genes, RPLP0 and PPIA (Applied Biosystems) were used as these have expression stability across CRC patient cohort samples
