# Supplementary material for: Estrogen Activation by Steroid Sulfatase Increases Colorectal Cancer Proliferation via GPER
Source: J Clin Endocrinol Metab. 2017 Sep 13;102(12):4435–47. doi: 10.1210/jc.2016-3716 (PMC5718700; doi:10.1210/jc.2016-3716)
Supplement: Supplementary file 3 [file jc.2016-3716.st3.docx]

| **Commercial Immunoblotting Antibodies** | | |
| --- | --- | --- |
| **Protein Target** | **Source** | **Catalogue No.** |
| HSD17B1 | Abcam | ab51045 |
| HSD17B2 | Protein Tech. | 10978-1-AP |
| HSD17B7 | Abnova | H00051478-M01 |
| HSD17B12 | Abnova | H00051144-M08 |
| ERa | Santa Cruz | sc-130072 |
| ERb | Abcam | ab288 |
| GPER | Santa Cruz | sc-48525-R |
| CTGF | Santa Cruz | L-20 |
| b-actin | Sigma-Aldrich | A5441 |
| goat anti-mouse | Santa Cruz | sc-2005 |
| goat anti-rabbit | Santa Cruz | sc-2004 |

**Supplemental Table 3:** Primary and secondary antibodies and suppliers used in the study.
