# Supplementary figures and images for: Estrogen Activation by Steroid Sulfatase Increases Colorectal Cancer Proliferation via GPER
Source: J Clin Endocrinol Metab. 2017 Sep 13;102(12):4435–47. doi: 10.1210/jc.2016-3716 (PMC5718700; doi:10.1210/jc.2016-3716)

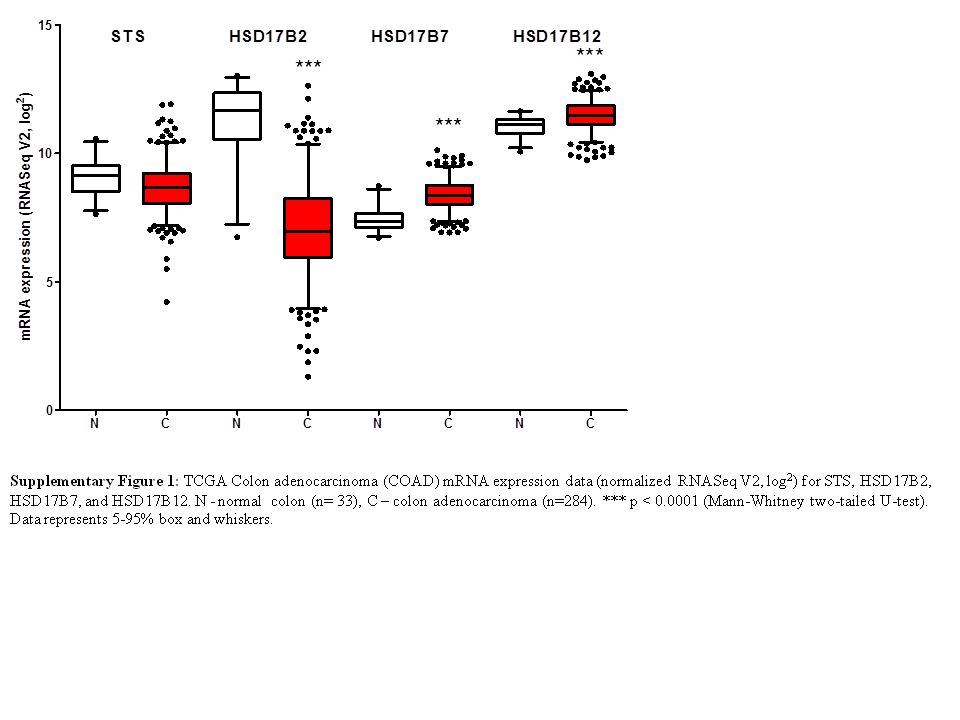

Supplement: Supplementary file 4 [file jc.2016-3716.sf1.TIF]

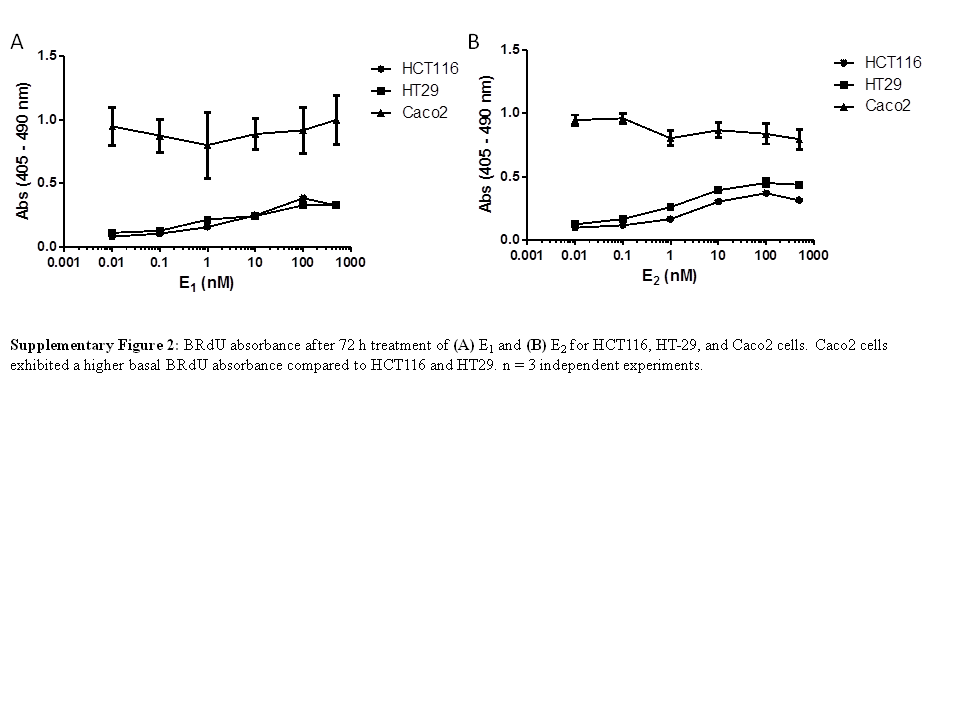

Supplement: Supplementary file 7 [file jc.2016-3716.sf2.TIF]

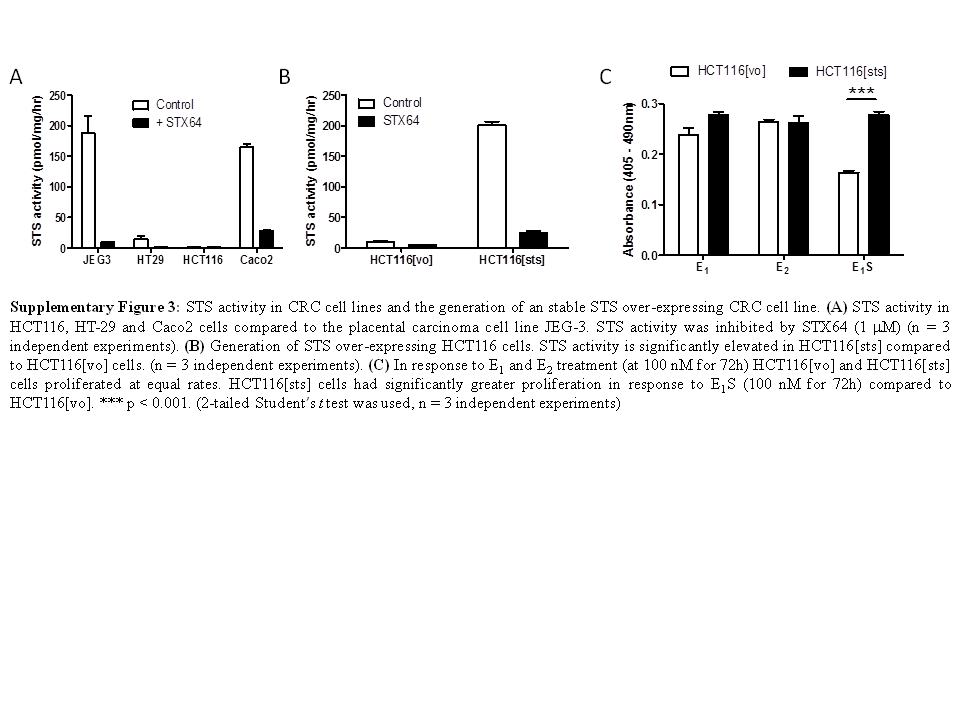

Supplement: Supplementary file 8 [file jc.2016-3716.sf3.TIF]

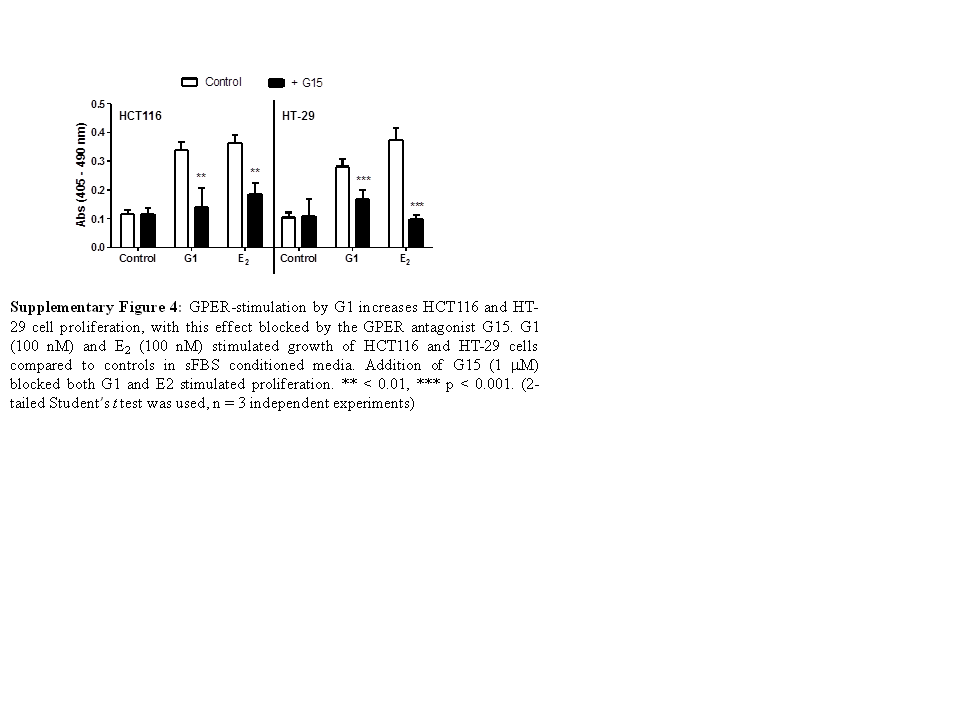

Supplement: Supplementary file 9 [file jc.2016-3716.sf4.TIF]

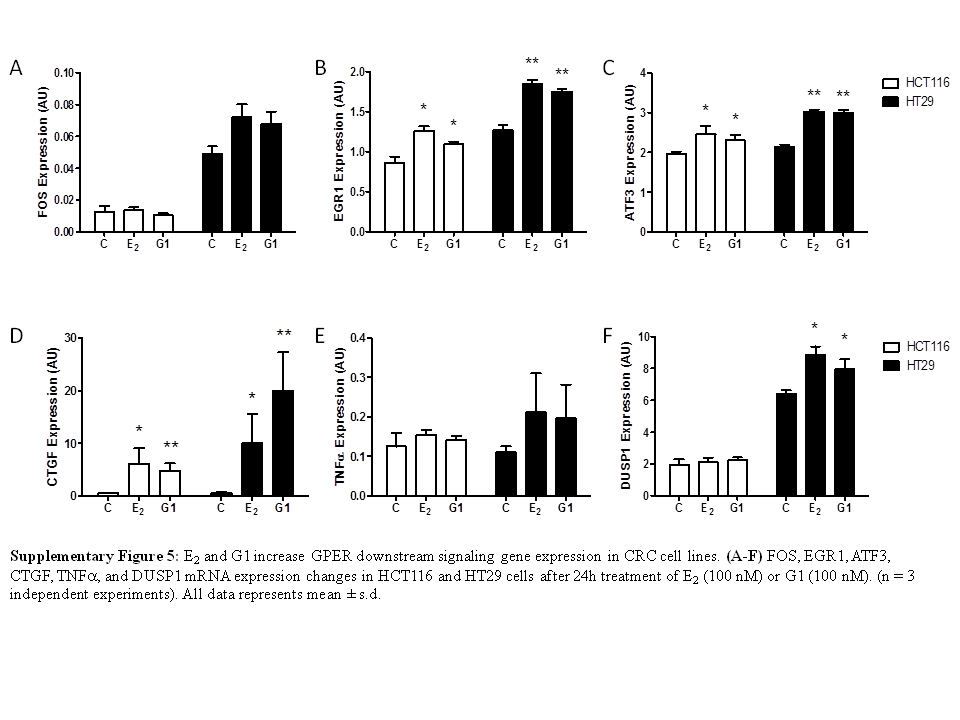

Supplement: Supplementary file 10 [file jc.2016-3716.sf5.TIF]

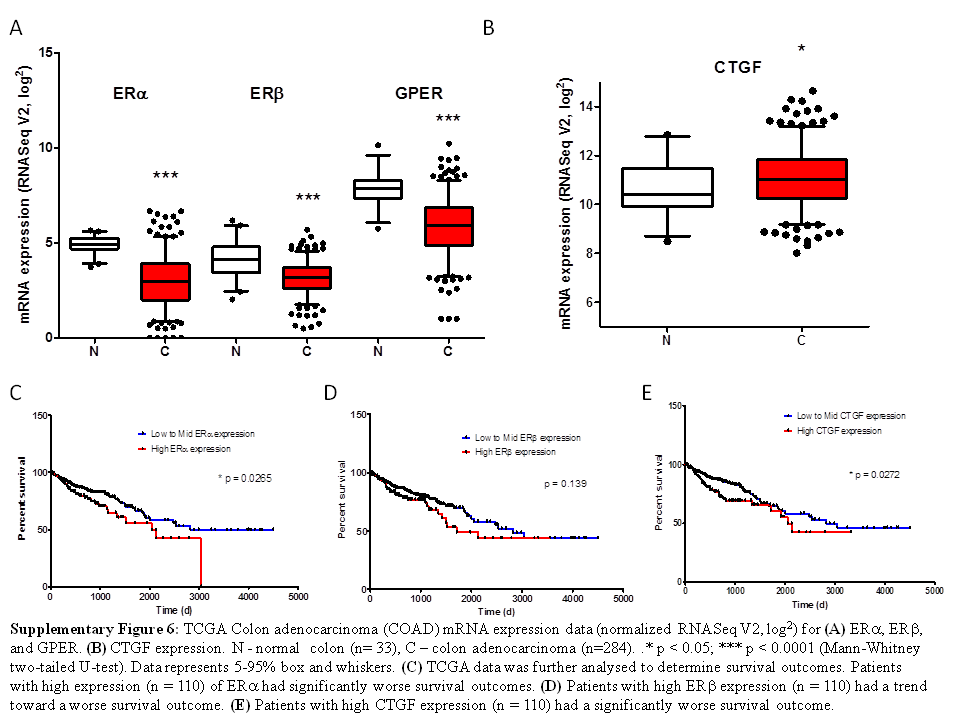

Supplement: Supplementary file 11 [file jc.2016-3716.sf6.TIF]

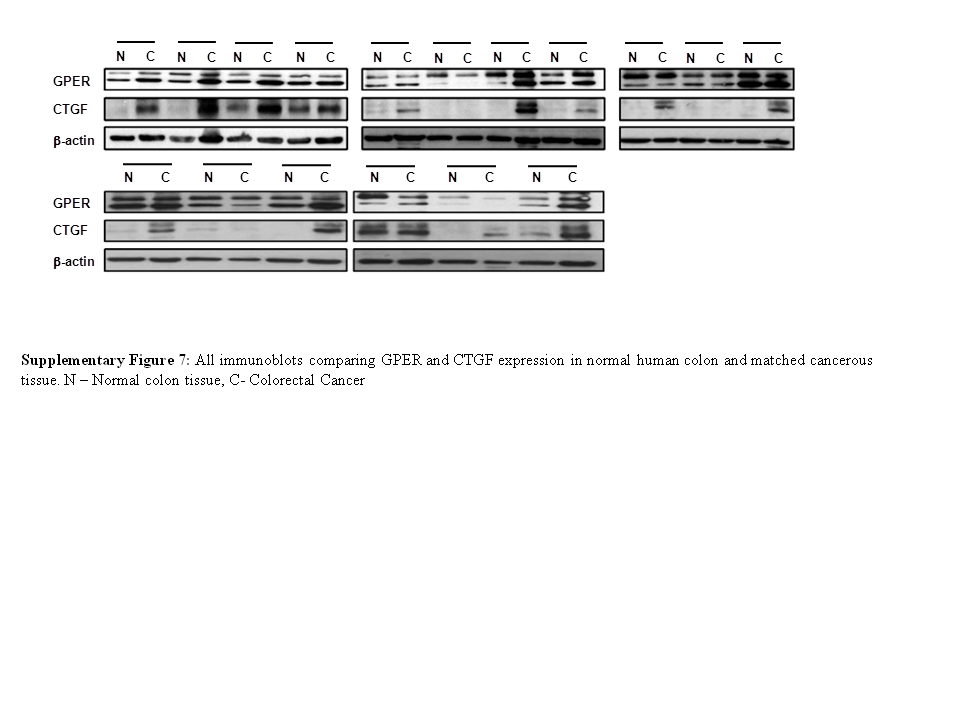

Supplement: Supplementary file 12 [file jc.2016-3716.sf7.TIF]
