# Supplementary material for: Estrogen Activation by Steroid Sulfatase Increases Colorectal Cancer Proliferation via GPER
Source: J Clin Endocrinol Metab. 2017 Sep 13;102(12):4435–47. doi: 10.1210/jc.2016-3716 (PMC5718700; doi:10.1210/jc.2016-3716)
Supplement: Supplementary file 5 [file jc.2016-3716.st4.docx]

| **Number of samples (patients)** | **HSD17B7 mRNA expression (dCT); n=84 (42)** | | **HSD17B12 mRNA expression (dCT); n=84 (42)** | | **HSD17B2 mRNA expression (dCT); n=93 (47)** | |
| --- | --- | --- | --- | --- | --- | --- |
|  | **Percentage change (95% Confidence Interval)** | **p-value** | **Percentage change (95% Confidence Interval)** | **p-value** | **Percentage change (95% Confidence Interval)** | **p-value** |
| **Cancer^a^** | -17.0 (-25.1, 8.9) | <0.001 | -58.1 (-82.9, -33.2) | <0.001 | 37.5 (-1.7, 76.8) | 0.061 |
| **Sex (male)^b^** | 18.6 (8.2, 29.0) | <0.001 | -25.1 (-0.9, 2.1) | 0.107 | 60.4 (18.6, 102.3) | 0.086 |
| **Age (years)** | 0.1 (-0.4, 6.5) | 0.612 | 0.6 (-0.9, 2.1) | 0.449 | -36.6 (-178, 1.04) | 0.611 |

^a^ reference is normal; ^b^ reference is female.

**Supplementary Table 4:** Results of random effects linear models investigating differences between HSD17B7 mRNA expression, HSD17B12 mRNA expression and HSD17B2 mRNA expression with status (normal/cancer) after adjustment for sex and age. The outcomes used in modeling were log transformed and estimates can be interpreted as approximate percentage changes.
