# Supplementary material for: Estrogen Activation by Steroid Sulfatase Increases Colorectal Cancer Proliferation via GPER
Source: J Clin Endocrinol Metab. 2017 Sep 13;102(12):4435–47. doi: 10.1210/jc.2016-3716 (PMC5718700; doi:10.1210/jc.2016-3716)
Supplement: Supplementary file 6 [file jc.2016-3716.st5.docx]

| **HSD17B2 mRNA Expression (dCt)** | **Female** | **Male** | **All** |
| --- | --- | --- | --- |
| Normal | 4.15 ± 0.44 | 2.99 ± 0.57 | 5.02 ± 0.40 |
| Cancer | 6.33 ± 0.76 | 5.25 ± 0.70 | 7.15 ± 0.53 |
|  |  |  |  |
| **HSD17B7 mRNA Expression (dCt)** | **Female** | **Male** | **All** |
| Normal | 8.56 ± 0.38 | 7.81 ± 0.38 | 9.34 ±0.29 |
| Cancer | 7.23± 0.24 | 6.56 ± 0.26 | 7.93 ± 0.18 |
|  |  |  |  |
| **HSD17B12 mRNA Expression (dCt)** | **Female** | **Male** | **All** |
| Normal | 4.39 ± 0.53 | 4.99 ± 0.62 | 3.73 ± 0.48 |
| Cancer | 2.76 ±0.34 | 3.06 ± 0.24 | 2.44 ± 0.46 |

**Supplementary Table 5:** HSD17B mRNA expression (dCt) in human colon tissue. n = 19 - 28.
